# Editorial

**Published:** 1841-02-13

**Authors:** 


THE MEDICAL EXAMINER.

PHILADELPHIA, FEBRUARY 13, 1841.

### DISEASES OF THE SKIN—PUBLIC BATHS.

Among the poorer classes of this and other large metropoles, cutaneous affections are very much neglected. Not immediately interfering with daily labour, and requiring for their treatment conveniences and appliances not to be found in the households of the poor, they are permitted to acquire a footing and progress that tell hurtfully upon the health of the community. It is hardly worth the while of physicians, connected with the charities of the city, to seek out and attempt to relieve this class of diseases, totally unprovided as they are with the proper means of treating them. The persevering employment of various descriptions of baths is an essential adjuvant in the successful management of cutaneous disorders. These are now entirely without the reach of poor patients. We offer as a suggestion to some of the opulent charities of Philadelphia, to connect with existing institutions, arrangements that will open to these patients the benefits of such a course of treatment. Baths may be established, tickets for which can be sold at a moderate price, or given in a reasonable amount, to the class of persons who now enjoy gratuitously medical relief. We may hereafter go more at length into this subject, contenting ourselves at present with throwing out the hint.

### EXPERIMENTS ON THE BODIES OF CRIMINALS.*

Louis and Orfila, by the anxiety they have shown to collect all the phenomena presented during the execution of criminals, and to study minutely the post mortem appearances, exhibit the importance they attach to such facts in a medico-legal point of view. The experiments of Orfila upon dead subjects, display only the mechanical injuries to which the suspended body is subjected; the organic alterations which would be produced in a living individual are of course wanting; we therefore deem it incumbent upon us to place upon record every circumstance which may serve to fill up this gap, and thus

* Continued from No. 5.

complete, as far as possible, the chain of evidence.

In the two latter of the cases with which we are occupied, the pulsations of the heart and of the artery at the wrist were noted shortly after the drop fell. The discrepancy is so great that we present the results in a tabular form.

| | PULSE. | | |
|---|---|---|---|
| | *Morris.* | | *Koebler.* |
| Minutes. | | Minutes. | |
| 1½ | 30, firm, rising. | | |
| 2½ | 40, strong. | | |
| | | 3 | 144 |
| 3½ | 64 | 3½ | 120 |
| | | 4 | 120 |
| 5 | 64, still irregular. | 5 | 150 |
| 5½ | 120, sudden rise. | | |
| | | 6 | 150, scarcely perceptible. |
| | | 6½ | 155 |
| 7 | too frequent to be counted. | 7 | 155 |
| 8 | 72, moderately strong. | 8 | imperceptible. |
| 8½ | suddenly ceased. | 8½ | no pulse at wrist. |
| | HEART. | | |
| | | 4 | sound obscure, rhythm perfect. |
| | | 4½ | less composed. |
| 5 | beats violently, 142. | 5 | too frequent to be counted. |
| | | 5½ | scarcely audible, very frequent. |
| 7 | 53 | 7 | 120 |
| | | 7½ | 132 |
| 8 | heart ceasing to beat. | | |
| 8½ | ventricle beats feebly. | | |
| 10 | no sound of heart or lungs. | 10 | 60 |
| | | 12 | 54 |
| | | 12½ | ceased. |

The pulse was accurately noted in both instances ; but it must be remembered that while the state of the heart was reported by Prof. Mitchell from immediate auscultation, the report of Dr. Duffee was derived from an examination by the hand, and must therefore be considered as less exact. We present the above table simply as a record of facts.

The post mortem examinations in the two first cases were very imperfect; that of Morris alone was complete; but unfortunately this was made at too late a period, and after the body had been subjected to numerous experiments, capable of altering the original condition of the organs. We cannot therefore deduce from it conclusions sufficiently rigorous to meet the wants of Orfila, and assist him in settling the great medico-legal questions upon which he is engaged.

Each examination however presents certain points of individual interest; and collectively they are remarkable for the absence of all those signs which are generally considered as indicative of death by suspension.

Opportunities of studying recent organs are not of frequent occurrence, and we regret that the above furnish us with so little of value. In one instance alone it was turned to profit. The eye was dissected and the tapetum found "quite distinct, as was also the spot of Sœmmering, but of a slightly greenish hue ;" in the others the occasion, although so rare, was unfortunately neglected.

In the two last cases the countenance was natural and placid ; in one a slight ecchymosis and abrasion existed at the posterior part of the neck opposite the knot; in the other no trace whatever of the cord could be detected. Priapism existed only in one instance, and ceased long before the body was removed from the scaffold : in both there was an emission from the urethra; this was subjected to microscopic examination in the case of Koebler, and found to consist of a few saline crystals suspended in mucus, but contained no spermatic animalculæ.

No rupture of the transverse ligament or dislocation of the vertebræ existed in either of the three cases, in the last, upon a minute examination of the neck, no trace of the cord could be detected; no injury to the larynx or trachea could be found, except that a light rose coloured circle was visible in the mucous membrane of the latter, immediately beneath the cricoid cartilage; the carotid arteries were perfectly sound; in a word, no signs existed calculated to point out the cause of death.

The appearances presented by the viscera were equally unsatisfactory; the left side of the brain in one case was more injected than the right, but the degree of injection was not positively anormal.

The *lungs* in all these cases were *entirely free from congestion;* it must be borne in mind however, that artificial respiration had been kept up at intervals, for two hours in each instance; how far this might have modified their appearance, by inducing a species of pulmonary circulation, we are not prepared to say; we merely present the fact as a singular one.

Another circumstance, not less remarkable, was observed in the case of Morris. The blood which was found in the heart contained *a large amount of air*. The same phenomena has been noticed in a case of death which occurred in a paroxysm of hydrophobia, during which the glottis was spasmodically closed, and the muscles of respiration acted violently: and we have been informed that a similar state of the blood was observed in three individuals who were simultaneously hanged some years since. The air could only be derived from the lungs themselves, and transmitted through the pulmonary vessels; may not this be of more frequent occurrence then is imagined, when death is accompanied by violent struggles? If so it is a sign not to be neglected.

On the whole, the results of the above investigation, although not devoid of interest, are unsatisfactory. We lay them before the profession, as calculated to afford assistance to future observers, and to aid in the compilation of future statistics.

